# Advances on the Understanding of the Origins of Synaptic Pathology in AD

**DOI:** 10.2174/138920207783769530

**Published:** 2007-12

**Authors:** Pascale Nathalie Lacor

**Affiliations:** Department of Neurobiology and Physiology, Northwestern University, Evanston, IL, USA

## Abstract

Although Alzheimer’s disease (AD) was first discovered a century ago, we are still facing a lack of definitive diagnosis during the patient’s lifetime and are unable to prescribe a curative treatment. However, the past 10 years have seen a “revamping” of the main hypothesis about AD pathogenesis and the hope to foresee possible treatment. AD is no longer considered an irreversible disease. A major refinement of the classic β-amyloid cascade describing amyloid fibrils as neurotoxins has been made to integrate the key scientific evidences demonstrating that the first pathological event occurring in AD early stages affects synaptic function and maintenance. A concept fully compatible with synapse loss being the best pathological correlate of AD rather than other described neuropathological hallmarks (amyloid plaques, neurofibrillary tangles or neuronal death). The notion that synaptic alterations might be reverted, thus offering a potential curability, was confirmed by immunotherapy experiments targeting β-amyloid protein in transgenic AD mice in which cognitive functions were improved despite no reduction in the amyloid plaques burden. The updated amyloid cascade now integrates the synapse failure triggered by soluble Aβ-oligomers. Still no consensus has been reached on the most toxic Aβ conformations, neither on their site of production nor on their extra- versus intra-cellular actions. Evidence shows that soluble Aβ oligomers or ADDLs bind selectively to neurons at their synaptic loci, and trigger major changes in synapse composition and morphology, which ultimately leads to dendritic spine loss. However, the exact mechanism is not yet fully understood but is suspected to involve some membrane receptor(s).

## INTRODUCTION

Alzheimer’s disease (AD) is the most common form of dementia in the elderly with half of all individuals over 85 years old developing it. AD is affecting over 4.5 million individuals in the United States and roughly more than 25 million individuals worldwide [1]. Even more individuals suffer from mild cognitive impairment (MCI), a disorder associated to memory loss thought to represent a transitional step between normal memory function and dementia [2,3]. AD, especially in its early stages, is a pathology of memory. This neurodegenerative disorder is characterized predominantly by initial alterations in recent memory, followed by progressive memory deficits, personality changes, and ultimately a complete loss of intellectual ability [2,4]. It is therefore not surprising that a large number of scientists have been trying to define AD etiology and to search for the devilish substances that are robbing people of their memories in the hope to develop treatments, which would prevent and combat destruction of cognitive functions and AD progression. Genetic and epidemiologic studies have established that early-onset AD is associated to dominantly inheritable mutations in at least one of three genes {APP, Presenilin-1 (PS1) and Presenilin-2 (PS2), see reviews [5,6] and references therein}. These mutations lead to increased production of amyloid β peptide (Aβ). Development of sporadic late-onset AD is associated to age, vascular factors, inflammation and diabetes as well as established susceptibility genes such as apolipoprotein E (APOE) [7-10] and the most recently discovered SORL1 [11]. These genes have been shown to increase Aβ deposition. Other genetic association studies in non-mendelian AD have proposed polymorphisms in various genes such as LRP1, MAPT, BDNF, IDE, A2M and ACE [8,12]. While the scientific community came to a universal acceptance about the role of the above-mentioned genes in AD, the basis for the early memory specificity of AD has not yet been completely revealed.

Hundred years ago, Dr. A. Alzheimer provided us with the first anatomical description of major brain neuropathological hallmarks found in the brain of a 55-year-old female patient, which he followed clinically for over 4 years before her death. His patient initially presented rapidly progressive impairment of memory associated to total helplessness and profound disorientation which evolved to general dementia. In her postmortem brain, Dr Alzheimer reported brain atrophy, presence of extracellular neuritic plaques, neurofibrillary tangles (NFTs) and massive neuronal loss (reviewed in [13]). Neuritic plaque and NFTs are the characteristic neuropathological signs that are still the main prerequisites the anatomopathologist uses during postmortem analysis to establish the diagnosis of AD according to various widely accepted criteria [14,15].

Other neuropathological hallmarks of AD include synapse loss, cholinergic and glutamatergic neuronal loss, gliosis and brain atrophy [16-19]. However, NFTs and amyloid deposits are constantly observed throughout the brain of elderly and centenarian individuals that had not previously met clinical criteria of dementia [20-23]. These lesions may be present for years without appreciable synaptic damage or neuronal loss [24] presumably this correlates with the absence of cognitive deficits and therefore calls into question whether these lesions represent an indication of a preclinical stage of AD. In non-demented cases, NFTs are usually restricted to the hippocampal formation, whereas their presence in association areas of the temporal neocortex matches the development of obvious clinical signs of dementia [15]. Conversely, neuritic plaques can be abundantly found in several neocortical areas in MCI patients and are poor predictors of AD severity as no correlation can be established between their density and spatial location (e.g., in limbic and cortical associative areas) with the degree of the disease [25].

We are still facing during the lifetime of the patients an uncertainty toward the clinical diagnosis of AD, which is based on standard diagnostic criteria established during interviews of the patient and family relatives, physical examination, cognitive function assessments and neuron-imaging tests. Most of the time, the neuroclinicians will properly diagnose (90% accuracy) the patient with “probable” AD when he is truly affected by moderate or severe AD [26]. However, MCI and mild dementia often are not detected. The lack of sensitivity in detection has stimulated research aiming at identifying AD biomarkers that will optimistically enter the clinical battery of tests available for the patients at an early time point in the disease progression. This would prove to be helpful for the physician to prescribe the current available treatment options that afford better delay in disease progression and therefore symptomatic benefits when administered early in the disease, ideally years before the dementia is diagnosed [27,28]. This review presents the remarkable progress that has been made in the understanding of the pathogenesis of AD, progress that was highly dependent on the development of new technologies in protein biochemistry, cellular and molecular biology, genetics and brain imaging.

This review will first describe: (1) facts that offered a “face-lift” to the main amyloid cascade hypothesis that now emphasizes the alteration of synaptic function and maintenance as an early stage of the disease, (2) evidence that pointed out to synapse dysfunction and loss in clinical studies and transgenic mice models of AD, (3) supporting data showing that soluble Aβ oligomers or ADDLs are the main neurotoxins which target particular synapses and (4) indications on how, possibly acting through putative receptors, downstream mechanisms might be used by soluble Aβ to trigger changes in spine composition and morphology.

## THE CLASSIC AMYLOID CASCADE HYPOTHESIS REVISITED 

The amyloid plaques and tangles, described by Alzheimer, are composed of insoluble extracellular deposits of fibrillar Aβ peptide and intraneuronal hyperphosphorylated tau aggregates respectively [29-32]. AD is now considered a prime example of protein misfolding disease [33]. For years, researchers have tried to determine if these neuropathological hallmarks are the cause of cognitive dysfunction observed in AD or if they just represented consequences of the real AD contributing causes. The prevailing constituent of the amyloid plaques, the fibrillar Aβ, was largely studied [34,35] and appears to decrease neuronal viability in rat primary hippocampal culture and to activate microglial cells [36,37]. At the time, Lorenzo and Yankner believed that preventing the fibrillation of Aβ rendered the molecules harmless [38].

There is an uninterrupted debate as to the relative importance of Aβ/plaques and tau/tangles to AD process. Initial studies by Yankner’s and Klein’s groups trying to establish a potential connection and causality between the two main hallmarks proposed that fibrillar Aβ induced the phosphorylation of tau *in vitro* [39,40]. In fact, Aβ deposition precedes the appearance of neurofibrillar tangles in human brains [41]. More recently, LaFerla’s group showed that Aβ immunotherapy, by reducing the amyloid plaque load lessen tau pathology in their triple transgenic animal model of AD, which would suggest that Aβ and amyloid plaques are responsible for tau pathology [42].

Aβ, as a monomeric form, is a naturally occurring highly hydrophobic peptide [43] that is generated *via *cleavage of the transmembrane glycoprotein APP (amyloid precursor protein) by 2 consecutive aspartyl proteases (see reviews [44-46] and references therein). Briefly the cleavage by β-site APP-cleaving enzyme (BACE) at the N-terminus of APP is the initiating step for amyloidogenesis. Subsequently, the (γ-secretase membrane complex containing presenilin (PS) amongst 3 other proteins cleaves APP in its transmembrane domain resulting in the extracellular release of the Aβ [47]. Although some of the Aβ can be generated in the endosomal vesicles or intermediate compartments and remained in the cell creating a pool of intracellular Aβ [48-50] that can also be released presynaptically. Released Aβ peptide afterward self-associates into either fibrils accumulating in amyloid plaques or oligomers, which are diffusible and soluble species [51-56]. PS-1 has loose substrate specificity. It does not only cleave other transmembrane proteins such as Notch and ErbB4, but it also cleaves APP at different positions resulting in the formation of several variants of Aβ which differ by the length of their carboxyl terminal (from 38 to 42 amino acids) [57,58]. The most common form of Aβ is Aβ_1-40,_ a 40 amino acids in length, which is normally secreted from cells and is suspected to be physiologically active [59]. The Aβ peptide 42 amino acids long (Aβ_1-42_) presents a more hydrophobic C-terminal, which provides a greater propensity to aggregate [60]. Its production is highly increased in AD patients where a shift in favor of the Aβ42 instead of Aβ40 is readily observable. Still, it is debated if there is a commonality or independence in the pathway leading Aβ to the aggregation into oligomers and fibrils [61,62]. It is particularly interesting here to remember that one of the first description of the core of amyloid plaques from AD and Down syndrome (DS) showed that it was composed of multimeric aggregates of Aβ (mainly dimers, trimers and tetramers) that are soluble in formic acid and identifiable by SDS-PAGE and HPLC. At this time, multimeric assemblies were believed to be part of the ongoing Aβ fibrillogenesis [63].

Integrating years of research on the deleterious effects of Aβ, Hardy and Higgins proposed in the 90’s the original “amyloid cascade hypothesis” [64]. This hypothesis described key steps that once dominated the train of scientific thinking for all research in the field of AD. They hypothesized that neurodegeneration occurring in AD is attributable to the accumulation of insoluble fibrillar Aβ species into amyloid plaques in the brain, which leads to the formation of neurofibrillary tangles subsequently followed by a massive neuronal loss leading to the observed dementia [64]. Some studies also suggest that Aβ disrupts calcium homeostasis [65], causes free radical generation [66] and various forms of peroxidative injury [67,68], and activates glia and microglia [69]. The dreadful role of Aβ was subsequently reaffirmed through a better understanding of the APP cleavage pathway and the genetic studies of APP and PS1 missense mutations (reviewed in [70-72]).

Afterward, other supports for this hypothesis were provided by the generation, in various laboratories, of transgenic AD mouse models (AD-Tg) carrying mutated genes or knock-in or knock-out genes that are involved in some familial forms of AD [73-76]. The most commonly used are the PDAPP, Tg2576, APPswe, APP/PS1 and 3xTg. These genetically modified mice develop progressive AD-type pathologies such as age-related elevated levels of Aβ and amyloid plaque deposition in the hippocampus and neocortex, tau hyperphosphorylation and variable synapse loss. For comprehension of their relevance to AD research, further details on the neuropathological characteristics of all created transgenic AD models can be found in the following reviews (for recent reviews [76-80]). AD-Tg mice also present age-dependent cognitive impairment in various learning and memory tasks [81-85]. Yet, these mice often do not present some of the other major neuropathological hallmarks of AD such as massive neurodegeneration or neurofibrillary tangles (NFTs) [77]. Neuronal loss, mostly in the hippocampus, was more frequently observed in the multiple transgenic mice [86-88]. Hitherto, these observations strengthened the hypothesis that AD neuropathology was resulting from the aggregation of Aβ and directly correlated Aβ aggregates with memory impairments.

It soon became evident that the original amyloid cascade hypothesis was not suitable to explain entirely the cellular and molecular mechanisms triggering the full range of neuropathologies associated with AD. The principal disappointment stems from the inconsistency between the amyloid plaque load in AD brains and the degree of AD dementia, an imperative correlation for a hypothesis asserting that memory loss is a direct result of fibrillar Aβ [89,90]. Although some reports showed significant correlations of plaque number with dementia [91,92], numerous studies have found only weak or no correlation between neocortical plaque densities and clinical severity measures [90,93-95]. A better correlation with cognitive impairment status shows up when the total Aβ burden is detected with Aβ specific antibodies [96]. Instead, densitometric measurement of synaptic population correlated much better with the cognitive performances of the AD patients [90,97]. Another group even proposed that distribution and abundance of NFTs are greatly associated with the clinical progression of AD [15].

The original amyloid cascade hypothesis also claimed that fibrillar Aβ ultimately killed neurons, suggesting an irreversibility of the disease. However, as mentioned above, the AD-Tg rarely present neuronal loss. In addition, surprising evidence obtained from a vaccination study in an AD-Tg refuted the irreversibility. Paul’s group passively immunized with a monoclonal antibody against Aβ some APP-Tg mice that have developed numerous amyloid plaques and presented memory impairments. Shortly after the vaccination, the mice showed a recovery of memory impairment despite the lack of reduction in the amyloid plaque load [98]. These results were supported in independent studies using a different monoclonal Aβ antibody and a different APP-Tg mice [99]. A major stumbling block to the amyloid hypothesis has arisen following these studies bringing contradictions to the initial thinking. The prompt reversal of memory loss observed in the vaccinated mice occurring despite no decrease in plaque load was totally inconsistent with a mechanism for memory loss dependent on neuronal death caused by amyloid fibrils. Researchers were faced with the following questions: How can memory loss be reversed if it is attributable to neuron death? In addition, how can memory recovery occur independently of Aβ plaque levels if memory loss is resulting from fibrillar Aβ species? Studies demonstrated that blockage of fibrillar Aβ formation by the protein clusterin also called ApoJ, which is highly expressed in amyloid plaques, did not prevent Aβ toxicity but to the contrary enhanced it [100]. A reevaluation of the amyloid cascade took into account the idea that memory failure derives from neuronal dysfunction rather than degeneration. AD has therefore ceased to be considered as an inescapable consequence of irreversible ageing.

New investigations were aimed at identifying the agent responsible for this fibril-free toxicity. Klein’s group characterized alternative Aβ assemblies and reported the presence of pathologically hidden neurotoxic assemblies of diffusible synthetic Aβ oligomers in the presence of clusterin [101], assemblies that can also occur in the absence of clusterin [102-105]. Non-fibrillar Aβ assemblies can be found in human brains of AD and Down syndrome (DS) patients prior to amyloid plaque formation [106,107] and are secreted naturally by cultured cells [108]. Concurrently, studies done in human brain demonstrated the presence of soluble Aβ concentrations which were highly correlated with disease severity [109,110], a correlation that has been confirmed with studies of MCI patients [111].

In 2002, Hardy and Selkoe proposed a revised amyloid cascade hypothesis incorporating as central role players the small soluble Aβ oligomers which are formed prior to fibrillar Aβ deposits. These oligomers are strongly believed to be the “memory-thieves” as they are particularly eager to impact synapse signaling, shape and stability before neuronal death occurs (description below). Indeed, many investigators have suggested that these oligomers constitute the main causative agent by interfering with neuroplasticity in the pathogenesis of AD [12,19,101,103,112,113-115]. This hypothesis really came forward after the demonstration that Aβ oligomers, which rapidly and selectively inhibit long-term potentiation (LTP), a classic experimental paradigm for memory and synaptic plasticity, did not seem to affect basal synaptic transmission, suggesting that the synaptic alteration observed was initially non-degenerative [56,101,116-119]. Recent findings have validated the revised hypothesis by demonstrating that soluble Aβ oligomers have clinical relevance. In fact, oligomers are strikingly elevated in AD-Tg [120] and, most significantly,in AD brain [121-123]. The updated amyloid cascade has already provided the foundation for pharmaceutical programs intending to develop drugs that would reduce Aβ production, aggregation or clearance [124] but it should also be noted that some of the events downstream of Aβ accumulation are still unsolved. It might also not be perfectly safe to develop therapeutics interfering with Aβ clearance that could affect dissociation of fibrillar Aβ into potentially synaptotoxic Aβ oligomers. Therapies that would eliminate Aβ pathology might prove to be insufficient if given at advanced disease stages [125]. In fact, recent clinical trials using vaccine against Aβ in AD patients have effectively decreased amyloid plaques burden, but not NFTs, and have conferred moderate clinical benefits [126-129]. Can the revised amyloid cascade survive without further re-examination? The basis for an “indirect amyloid hypothesis” has recently come to light. In this new hypothesis, leaders in the pharmaceutical industry have pointed out the need for further research that will not exclusively revolve around the generation and accumulation of Aβ but take into account parallel events and therefore provide alternative targets (e.g., tau protein and apolipoprotein E) [130]. Additional basic research studies need to be conducted to help the development of treatment that will afford cognitive improvements to AD patients and ultimately will stop Aβ-dependent pathologies such as tau accumulation, oxidative stress and neurodegeneration. These treatments would ideally provide help to patients at whatever stages of the diagnosis they are found in while progress is made in the development of reliable brain imaging techniques and early detection of AD markers such as some of the recent nanotechnology based assay developed to measure soluble Aβ in the CSF [131].

## SYNAPSE PATHOLOGY IN AD AND AD-TRANSGENIC ANIMALS

Among the series of abnormal alterations in the AD brain, the most significant proximal events are loss of synapses (pre- and post-synaptic compartments) and neuronal cell death. The majority of original reports on organized neuronal networks, structure-wise, were obtained using the Golgi impregnation of cerebral cortex sles obtained from deceased AD patients. Despite the known technical limitations of this method, such as a non-selective and poor sling of neurons (2-10% of the neurons being labeled), it provided the first substantiation of dendritic damage with marked spine loss in AD [132]. More recently, proteomic and genomic studies, conducted in humans and AD-Tg, have confirmed the downregulation of genes involved in synaptic maintenance and function (e.g., signal transduction, synaptic vesicle machinery, exo- and endocytosis related genes, calcium binding and cytoskeleton) convoyed by overexpression of cellular stress response molecules early in the disease progression [133-141]. Beside the fact that Aβ can impact the expression of synaptic proteins and regulate synaptic mechanisms involved in memory processing, it is also possible that synaptic dysfunction in AD results, at least in part, from inherited variations in synaptic genes.

Dendritic spines are the post-synaptic compartment of the synapses and are virtually where all excitatory afferences make contacts on hippocampal and cortical pyramidal cells in adult brain [142]. Dendritic spines therefore reliably reflect the number of synapses and are often used as an index for synaptogenesis and synaptosis [143]. They also represent sites where significant changes are made to accompany functional and structural plasticity [144,145].

Dramatic loss of synapses and dendritic spines in AD was confirmed by morphometric analysis both in autopsied and biopsied AD samples. More than 50% spine loss was observable in hippocampus and neocortex [146,147] and synapse loss was noticeable on surviving neurons as well [97].

Development of specific antibodies for synaptic markers has provided new tools to assess the synapse loss in AD. Levels of synaptophysin (a protein highly expressed in presynaptic terminals) quantified within key memory centers of the brain (e.g., frontal cortex and hippocampus) were highly decreased and strongly correlated with the degree of memory impairment assessed by cognitive tests before the AD patient’s death [90,148-150]. Synapse loss was not restricted to areas surrounding plaque deposition [151].

In AD-Tg, no correlation was found either between plaque load and synapse loss but synapse loss correlated well with increased levels of soluble Aβ [86,152-154] particularly in regions highly involved with memory processing such as the perforant path input from the entorhinal cortex to the dentate gyrus. Synapse loss seems to be the first morphological sign in the neuronal dysfunction and as depicted in the 3xTg, synapse loss and disrupted LTP appeared prior to plaque deposition, formation of tangles or cell death.

Nonetheless, synapse loss seems to appear after the surfacing of cognitive impairment which points to the possibility that initial events rather implicate “synaptic failure” prior to the established “synaptic slaughter” and neuronal loss in AD [19,155,156].

Recently, quantitative immunoblotting experiments aimed at measuring synaptophysin (SVP), synaptotagmin (STG) and drebrin (DRB) levels [157] in tissues sles from the Religious Orders Study (ROS) [158] revealed differential regulation of these markers in 5 neocortical regions both in MCI and mild/moderate or severe AD. Levels of SVP selectively decreased in the superior temporal and inferior parietal cortex of severe AD and correlated with MMSE scores. However, levels of STG were unaffected. DRB, an f-actin binding protein localized in dendritic spine heads of excitatory synapses which plays a role in synaptic plasticity by regulating spine morphogenesis and formation of postsynaptic densities [159,160], was decreased depending on the investigators from 40 to 90% in cortical regions [161,162] and ~70-80% in hippocampus of patients with severe AD [163]. DRB levels in the anterior cingulated, superior temporal and visual cortex correlated with MMSE and its decrease is observable in MCI as well [157]. A shift in drebrin represents a good indicator of changes in dendritic spine density. Progression of cognitive decline during the clinical course of AD may be related to a loss of excitatory postsynaptic contacts within vulnerable brain regions. If NFT formation plays a role in postsynaptic marker loss remains an open question [134,164]. NFT deposition might be associated with decreased transsynaptic efficacy in some cortical region. Increase of DRB was found in superior frontal cortex in MCI subjects with Braak stage II-IV (high limbic stage), consistent with other observations reporting increase in MAP2 throughout cortex of non- or mild-demented subjects with Braak stage IV [165]. At this stage, NFTs are relatively sparse in regions involved in executive functions (frontal cortex) but are abundant in regions involved in associative behaviors such as memory and language (temporal cortex) [15]. Therefore, increase in postsynaptic markers in specific brain regions may represent a dendritic structural reorganization to preserve cortical neurotransmission despite the mounting neuropathological damage. Significantly, there is an upregulation of ChAT activity in the same brain regions in MCI from the ROS cohort [166].

Studies above demonstrate that initial plasticity responses in some brain regions contribute to functional preservation at least during early stage of AD. A model for synapse morphology alterations during AD progression was actually proposed by Scheff and Price [167]. AD synapse pathology begins with a slight loss of synapses resulting from cholinergic and glutamatergic deafferentation [168,169], which is then accompanied by a decrease in the number of dendritic spines. Over time, new synaptic contacts are formed by sprouting from adjacent afferent terminals, which occurs concurrently with the enlargement of pre-existing synapses in the attempt to compensate for early synapse loss. However, the compensatory mechanisms are eventually overwhelmed as the disease progresses, resulting in a net decrease in synaptic contact area [167]. The loss of synaptic contact area interrupts functional neural circuits, resulting in cognitive deficits [170]. Further evidences of Aβ-associated synaptic pathology come from studies of patients with DS and AD-Tg. 

DS patients present cognitive deficits and early onset AD [171]. In these patients, overexpression of APP resulting from an extra copy of chromosome 21 induces increase in amyloid levels associated to a decrease in spine density. The “surviving” spines are morphologically different, rather larger or thinner, than spines expressed in non-demented subjects [147,172,173]. Decreased levels of dendritic spine marker proteins such as synaptosomal associated protein 27 [174] and F-actin bundling proteins (such as drebrin and moesin) [161,175,176] in DS patients also suggest synaptic defects consistent with the memory deficits seen in patients, and with abnormalities in synaptic plasticity such as LTP seen in DS-Tg model mice [177,178].

Conflicting information on synaptic pathology have been gathered throughout the years in various engineered AD-Tg models, [179] but most of the lines present some degree of changes in synaptic marker expression [180-184]. More recently, using the latest *in vivo* imaging techniques (i.e, DiOlistic method coupled to intravital multiphoton microscopy and high throughput morphometric analysis or PET & MRI), marked dendritic spines loss was reported in specific brain regions [185-189]. Shelansky’s group first showed using fluorescent dyes that the postsynaptic sites of the synapses, the dendritic spines, were dystrophic and less dense in AD models than age-matched control sles and, in support of neurotoxicity induced by soluble Aβ species, these deficits were not confined to plaque deposited regions [186]. Interestingly, dendrites traveling through plaques can still harbor spines but their morphology is different from that of the amyloid-stained dystrophic neurites surrounding them. In AD-Tg, loss of synaptic terminals and pre- and post-synaptic markers is rather convincing [88,181,190] and is also consistent with decreases in transcripts of multiple synaptic genes [137,155]. In Ts65Dn, a mouse model of DS with severe amyloid deposition, dendritic abnormalities are characterized by less spinous and less branched basal dendrites of frontal cortex pyramidal cells compared to diploid controls [191]. 

However, there is still a lack of consensus on the relationship between plaque load and synapse loss in AD-Tg. Some investigators proposed that synapse loss occurs to certain degree independently from extracellular amyloid deposition (happening prior to or at distance from it) and correlates well with increasing levels of soluble Aβ [152,154,185, 188,192,193]. Other groups reported, using *in vivo* measurement, a higher decrease in dendritic spine density in or nearby amyloid plaques, emphasizing that fibrillar amyloid deposition leads to local synaptic abnormalities and breakage of neuronal branches [187,194-196].

This appears to be in contradiction with the “soluble Aβ oligomer hypothesis” but it should be considered that plaques might possibly be a reservoir for toxic soluble oligomeric forms of Aβ [53]. It should also be noted that, soluble forms of Aβ have not yet been made readily observable for in situ microscopy particularly because of their diffusible nature and the lack of dye/reagents usable for imaging. While they are cryptic to the neuropathologist, their levels can be estimated by biochemical techniques. 

Novel technical developments, combining high resolution imaging techniques and the manufacture of high affinity amyloid-binding compounds (e.g., “Pittsburgh Compound B”), will certainly enable us soon to determine better the spatial resolution of the amyloid plaques (neuritic or diffuse) in living subjects. It is now possible to detect cerebral amyloid deposits by *in vivo* PET detection [197-199] and such approaches have been successful not only in the AD-Tg models but also in living human subjects giving hope for future diagnostic tools in AD field [200]. 

## SOLUBLE Aβ-OLIGOMERS AS IDENTIFIED GAIN-OF-FUNCTION SYNAPTOTOXINS

After many years of research on amyloid toxicity, it has become evident that, even if the harmless monomers of Aβ require to self-associate in order to become neurotoxic, the ultimate aggregational state (fibrils) might not be carrying all or even any of the neurotoxic effects [12,113,201,202]. This section will review evidences in favor of the soluble Aβ-induced synaptic failure.

Aβ1-42 monomer, a peptide with a better propensity for aggregation than Aβ1-40, can adopt different multimeric assemblies communally termed “soluble Aβ” with differently conformed soluble Aβ aggregates exhibiting most likely a large spectrum of toxicity and playing a fundamental role in AD pathogenesis [56,203]. Some reports in the literature describe better than others the type of Aβ species studied while other apply freshly dissolved Aβ1-40/42 without a clear definition of the assembled status reached in their experimental conditions. Comparison between studies therefore need to consider this carefully because the aggregated forms attained depend on the concentration of Aβ and experimental environment and are extremely different when started from Aβ1-40 or Aβ1-42 [204]. In fact, it has become evident that Aβ1-40 and Aβ1-42 use different oligomerization and fibrillation pathways and while Aβ1-42 creates multiples Aβ assemblies from 3-mers to protofibrils, Aβ1-40 mostly appears as 1-4mers prior to become fibrils [61,62]. The term “soluble oligomer” has a more general use as it takes in all soluble forms of Aβ just after its production by the cells or the soluble aqueous fractions obtained either from brain extracts or from the synthetic Aβ peptide preparations.

Investigating the role of soluble oligomers in the pathogenesis of AD presents difficulties mostly in distinguishing these soluble metastable assemblies from mature fibrils and soluble monomers. Detection methods comprise mainly SDS gel electrophoresis, gel filtration, circular dichroism, light scattering, size exclusion chromatography (SEC), atomic force microscopy (AFM) and electron microscopy. Further characterization was recently facilitated by using the PICUP method which “stabilizes” the oligomeric species in their adopted molecular organization [205]. Recent immuno- and nanotechnology-based assays are taking advantage of the latest development of antibodies raised against particular Aβ conformations by Klein’s lab (M94, M71, M69, NUs) and Glabe’s lab (A11), [123,102,206]. The aggregation of Aβ generates a range of toxic intermediary oligomeric assemblies (see reviews [56,207]) for instance small/low-n oligomers (dimers, trimers; [53,108], globular oligomers/ ADDLs (Aβ-Derived Diffusible Ligands – 3/4-24mers; [101-105,208], Globulomers (38/48kDa;[209]), Aβ oligomers/AβOs (15-20mers; [206,210]), Aβ*56 (dodecamers; [211]), ADDL-like spherical assemblies [212,213], annular assemblies (Doughnut-like structures) [214] and protofibrils/high-n oligomers (string of oligomers, [51,61,201,215]). It is still debated if any of these intermediary forms are on the pathway to fibrils [54,55,61,201,202,216-218]. Further studies will provide possible distinction between these oligomeric assemblies concerning their biological activities and their neurotoxic properties. In view of so many different conformations, it is legitimate to expect that different pathways might be activated through potential binding to membrane receptor(s) or by a direct damage to the neuronal membrane.

### Presence of Soluble Aβ in Human and AD-Tg

In the 90’s, six major studies proposed that non-fibrillar Aβ42 was actually the first species to deposit extracellularly in the cerebral cortex of AD patients [106,110,219-222]. Small oligomers of Aβ were present in SDS extracts of AD-affected blood vessels but believed to be indicators of ongoing fibrillogenesis. Roher’s group isolated water-soluble Aβ oligomers from normal and AD brains using a centricon fractionation and demonstrated that soluble Aβ levels and particularly the Aβ42 form were greatly increased in AD patients compared to controls, but again they considered these species to be intermediates en route to formation of toxic amyloid fibrils. Younkin’s group confirmed presence of soluble Aβ species in AD brains [223,224]. Not long after, Roher suggested that the water soluble dimeric species are more neurotoxic than high molecular weight fibrillar species of Aβ40 [225] whereas recent studies indicated that dodecamers from Aβ42 are more potent neurotoxins [114,115,122,202,211,226].

Few findings have validated the revised amyloid hypothesis by demonstrating that soluble Aβ oligomers have clinical relevance. Detergent-free aqueous extracts of AD brains have shown manifold increase of soluble forms of Aβ when compared to extracts of age-matched non-demented controls [121,122]. Most prominent species in these AD brain have an isoelectric point of 5.6 and a molecular weight around 56kDa equivalent to an assembly of 12 monomers [121]. Similarly, the presence of an Aβ species, named Aβ*56 (for 56kDa), was found in Tg2576 mice at a young age, prior to amyloid plaque deposition and obvious neuronal loss. In this study, trimers and hexamers were disregarded as potential synaptotoxic as their presence in the brain occurred prior to cognitive deficits. Isolated Aβ*56 from the Tg2576 mice was subsequently injected in rats and triggered impairment suggesting its involvement in cognitive decline [211]. Aβ*56 was rather more detrimental in behavioral paradigms than fibrillar Aβ [227]. 

The* in situ* distribution of soluble Aβ oligomers in human brains was detected by either A11 from Glabe’s lab, which does not recognized either 2- to 4-mers or Aβ fibrils, or ADDL-raised antibodies from our lab, recognizing oligomers [122,123,206]. Oligomer-immunoreactive plaques were diffuse and did not co-distribute with Thioflavin S-positive dense-core plaques, which contain fibrillar Aβ. Using our oligomer-selective antibody, we described a diffuse immunostaining surrounding neuronal cell bodies (perineuronal labeling) rather than intracellular deposits, which could be interpreted as apparent oligomer binding within dendritic arbors [122] reminiscent of the diffuse synaptic-type deposits observed in prion-associated diseases [228]. 

Using anti-globulomer antibodies (8F5 and 5598), soluble Aβ can be found in periphery of plaques in AD and AD-Tg as well as prior to mature amyloid plaques [209]. In AD brains, accumulation of soluble Aβ was detected, using our oligomer-specific antibodies in an immunoblot assay, in region implicated in memory functions such as the frontal cortex but not in the cerebellum which is generally preserved in AD [121-123]. Increased levels of soluble Aβ was recently reported in biopsied temporal cortex from patients suffering from traumatic brain injury [229] which could explain some of the cognitive disturbances present in these patients [230].

Oligomers are also strikingly elevated in various AD-Tg in an age-, transgene- and region-specific manner [42,120,211,231]. In the 3xTg mice age-dependent accumulation of soluble oligomers was detected using the two main oligomeric antibodies available [102,123,206]. Moreover, the results of immunization trials referred to above strengthened earlier studies demonstrating significant correlations between levels of soluble Aβ species, cognitive deficits and synapse loss in humans affected with AD and in various transgenic mouse models of AD.

### Soluble Aβ Oligomers Obtained from Synthetic Aβ1-42 Preparations or from Conditioned Media of hAPP Over-Expressing Cells 

Klein’s group has discovered the presence of pathologically hidden neurotoxic assemblies of Aβ named ADDLs. They are small diffusible globular metastable assemblies formed from synthetic Aβ1-42 monomers in the presence or absence of clusterin [101,102,104]. ADDLs are metastable SDS-resistant preparations that range from trimers to 24-mers and are temperature sensitive, with 12-mers or tetramers predominating when solutions are put at 37°C or 4°C respectively [101,104]. ADDLs were injected into rabbits and mice and successfully triggered the production of ADDL-specific antibodies which were afterward shown to subdue ADDL toxicity in cell culture [102,123].

Simultaneously, Selkoe and Walsh groups have engineered a CHO cell line, 7PA2, in which the human APP was inserted and they have collected several oligomeric species secreted from these cells. Oligomeric species contained in the conditioned media and separated by SEC were SDS-stable dimers and trimers [56,108,118,232,233].

At nanomolar concentrations, soluble Aβ oligomers, synthetically prepared or endogenously released from 7PA2 cells caused severe impairment of long-term potentiation (LTP) as well as the reversal of long-term depression (LTD). This effect was observed after *in vivo* microinjection of the oligomers or in *ex vivo* experiments using hippocampal slices demonstrating their neurotoxic activities [19,101,113, 116,118,234].

If monomers contained in the oligomeric solutions were eliminated by digestion with the insulin-degrading enzyme, inhibition of LTP was not affected, reinforcing the idea that oligomer assemblies are responsible for LTP blockage. It would indicate that the net synaptic activity is shifted toward inhibition and strongly suggest instability in synaptic composition and morphology [103,122]. Counterpart studies proposed that rod-like assemblies known as protofibrils could interfere with proper LTP [51,235]. These observations confirmed that soluble Aβ oligomers could directly impair synaptic plasticity and memory formation in agreement with studies demonstrating that LTP was impaired in some AD-Tg (e.g. PD-APP or 3xTg) before the development of Aβ deposits [86,236]. Such impairment of synaptic plasticity may also account for plaque-independent cognitive failure seen in hAPP-Tg mice [152,192,237,238], which accumulate soluble oligomers as mentioned above [120]. Soluble oligomers have also been found to interfere with memory functions tested by widely used behavioral tests specific for memory assessment in rodents [211,239] and their “inactivation” by NAB61, an Aβ oligomer-sensitive antibody, ameliorates memory functions [240]. The detrimental effect of oligomers on LTP and impaired cognitive performance can therefore be annulled by specific anti-Aβ antibodies [56, 241]. Blockage of LTP by ADDLs suggested a mechanism by which the neuronal dysfunction may present itself particularly in the view of their abundance in early stages both in AD and AD-Tg brains.

*In vitro* studies, conducted in Klein’s group, showed that within 5 minutes both synthetic ADDLs and soluble aqueous extracts of human AD brain containing oligomers bind to hippocampal and cortical, but not cerebellar, neurons with a pattern that is punctate-like reminiscent of hot-spots distribution adopted by surface receptors [121,122]. In these highly differentiated and synapse enriched-cultures, ADDLs bind specifically to neurons expressing glutamatergic receptors but failed to bind to GABAergic neurons (GAD-positive cells) or to astrocytes (GFAP-positive cells) [122,226]. Interestingly, when added to primary hippocampal cultures, the CSF of AD patients, but not age-matched controls, displayed similar hot-spot distribution pattern of ADDL-IR punctas [122]. Taking advantages of a sophisticated nanotechnology assay, known as BioBarcode, it was demonstrated that in fact ADDLs accumulate in CSF of AD patients giving hope for the development of a biomarker for AD diagnostic [131]. 

In order to advance our understanding of the mandatory Aβ conformation that triggers neurotoxicity, we studied the punctate distribution of fractionated aqueous AD extracts and synthetically prepared ADDLs. Centricon filter fractionation of AD extracts showed that binding activity resided in oligomers of molecular weights comprised between 10 and 100 kDa, consistent with the 2D native electrophoresis characterization reporting dodecamers as the most prominent species in AD brain [121]. Similarly, when synthetic ADDL species were separated by centricon fractionation or HPLC size-exclusion chromatography, the fractions carrying the binding abilities were comprised between 50 and 100kDa consistent with the size of 12-mers and larger species, but not with protofibrils. No binding was obtained either from fractions containing monomers and small oligomers (2-,3- and 4-mers) [122,226]. This seems inconsistent with the synaptotoxic effect afforded by the exogenously applied naturally secreted oligomers (mostly dimers and trimers) [118,242] but in agreement with the toxicity afforded by larger oligomers (12-24mers) [243]. This discrepancy calls for further studies. One reason for this divergence that comes to mind is the possibility of a differential targeting location one being extracellular and at the synapses while the other might act intracellularly after endocytosis of the Aβ species for example. It is also possible that both species categories trigger similar neurotoxic effect by different mechanisms. Ultimately, the difference in concentrations of the oligomeric species present in the two different preparations can be held responsible, as recently proposed by Halpain’s group [244]. Subsequent studies revealed that oligomeric Aβ_42_ were more neurotoxic than monomeric and fibrillar preparations at sub-micromolar concentrations [212]. Although Aβ1-42 has been reported to accumulate intracellularly in AD and AD-Tg within neuritic processes and synaptic terminals [86,245, 246], ADDL immunoreactivity on neurons was almost solely found at cell surfaces, even after cell permeabilization protocols.

Binding of ADDLs most probably occurs through attachment to membrane protein(s) because ADDL punctas are lost when cells have been trypsinized [101]. Despite the fact that many potential membrane receptors have been proposed [247], the identity of the soluble Aβ oligomers binding protein(s) is still unrevealed. Ongoing studies in search of the receptor(s) are building on initial ligand overlay assays, which monitor protein interactions, and yielded to a couple of membrane-associated binding partners (p140 and p260) from hippocampus and cortex [121].

The distribution of ADDL punctas on dendritic arbors, as depicted in Fig. (**1**), was observed either with synthetic ADDLs, AD brain-derived soluble oligomers or CSF suggesting that ADDLs might bind to synapses, as would be anticipated for a molecule that could disrupt LTP and LTD. Indeed, over 90% of the neuronal-targeted ADDLs do colocalize with PSD-95, a postsynaptic protein implicated in organization, function and plasticity of excitatory synapses [248], while 50% of all synapses are targeted by ADDLs [122]. Using similar quantitative analysis, Busciglio’s group confirmed the synaptic binding of ADDLs and AβOs (~90kDa species) on human cortical neurons [243], ADDLs being more synaptic than AβOs. In agreement with our data, specific binding to hippocampal neurons but not astrocytes was obtained with the globulomers [209]. We also confirmed that ADDL-binding spots are abundantly present at post-synaptic sites. ADDLs specifically colocalize with various synaptic markers highly enriched in the post-synaptic sites such as PSD-95, αCamKII, Arc, spinophilin and drebrin but were found apposed to the presynaptic marker synaptophysin [226,249,250]. Post-synaptic localization of ADDLs indicated by microscopy was completed by both ELISA assay and subfractionation of the synaptic junctional complexes. By ELISA and subcellular fractionation, ADDLs were associated with PSD rather than presynaptic active zones [226,251]. Further confirmations came from immunoprecipitation experiments of ADDL-treated cortical synaptosomes. Detergent extraction of the synaptosomes yielded an ADDL-binding complex that was in essence postsynaptic as attested by the presence of PSD-95 and the absence of syntaxin, a presynaptic active-zone protein. Further extraction by sarkosyl and SDS detergents released PSD-95 as well as some fundamental subunits of the NMDA receptors. This strongly supports the ability of ADDLs to attack synapses where they can initiate local synaptic dysfunction.

The synaptic selectivity for a subpopulation of neurons needs to be further characterized. It is suspected that some synaptic constituents or the combination of some of them make some synapse particularly vulnerable. While PSD-95 is expressed ubiquitously in synapses throughout the brain, other postsynaptic constituents might be differently represented in assorted synapses. This might in turn give variability to the subunits composition of glutamate receptors, to membrane receptor interactions, to the abundance of some scaffolding proteins (e.g., SynGAP, homer, shank, etc…) as well as to the abundance of molecules of the signaling cascade (e.g., kinases/phosphatases and RhoGTPases).

As mentioned above, Aβ can be produce intracellularly and aggregate in a variety of subcellular compartments, including the endoplasmic reticulum (ER), trans-Golgi network and lysosomes in AD and DS patients [252]. It has been proposed by some investigators that intracellular accumulation might be an event occurring prior to extracellular deposition and suggests that extracellular deposition results from release of intracellular Aβ by “leaky” or “dying” neurons [246]. The role of intracellular Aβ in synapse toxicity is still the object of controversy. In the 3xTg and Arcβ mice (expressing the artic mutation), deficits in synaptic plasticity, learning, and memory appear to be associated with the buildup of intraneuronal Aβ as they occur prior to plaque formation (see [253] for recent review). Appealing data were obtained from the immunization with anti-oligomer antibodies which demonstrated protection against Aβ detrimental effect at synapses after their internalization [254]. Clearance of extracellular Aβ occurred before the clearance of intracellular Aβ in another model [255]. These studies indicate the importance of intracellular Aβ [256] but raise the following questions: what are the respective contribution of extra- and intracellular Aβ oligomers in the pathogenesis of AD? and what is the source of the intraneuronal Aβ? does it originate from retention and subsequent aggregation of intracellularly generated Aβ or does it come from the reuptake of extracellular Aβ?

In fact, if exposure to ADDLs significantly affects synaptic morphology and composition which are known to influence several key aspects of synaptic transmission, it is fair to believe that ADDLs are the initiators of early cognitive impairment observed in AD patients and participate throughout the progression of the cognitive impairments. Therefore, a “memory thief” molecule would be considered a good synaptotoxin only when enough synapses of a determined memory-involved network are altered by it and both synaptic compensatory homeostatic mechanism and synaptic reserve becomes overwhelmed [257]. Remarkably, we have recently reported that ADDLs hold biological and physiological uniqueness that would be anticipated for such a synaptotoxin liable for AD dementia. In fact, ADDLs have been found to trigger changes in dendritic spine composition, density and structure [226,250,258,259]. 

We initially showed that when ADDLs are bound at synaptic sites, they can trigger some synaptic events such as the over-expression of Arc [122,260], a synaptic immediate-early gene product implicated in LTP and for which the overexpression has been linked to dysfunctional learning [261,262]. Upon binding to mature hippocampal neurons, ADDLs also promoted a rapid decrease in membrane expression of memory-related receptors (NMDAR, EphB2 and InsR) that can not be attributed to spine loss but rather to a local synaptic alteration [226,259,263]. Using different Aβ preparations that are expected to yielded reasonable amount of low molecular weight oligomers, a major loss of postsynaptic density protein, PSD95 [180,264], and synaptic receptors, NMDA-R and AMRA-R [180,265-267], was also described. Continued exposure to ADDLs resulted in abnormal spine morphology [226], with a temporary induction of long thin spines reminiscent of the morphology found in mental retardation, deafferentation, and prionoses [173]. We have observed a transient increase in synaptic boutons size at time when some dendritic spines were elongated [114,249] which would suggest that ADDLs might also be involved in reactive synaptogenesis compatible with the concept that early stage synapse degeneration in AD includes adaptative enlargement of presynaptic terminals [165,268]. Synaptic binding of ADDLs is associated to alterations of the F-actin cytoskeleton and the microtubule network as respectively demonstrated by the redistribution and decrease of drebrin protein [226] and hyperphosphorylation of tau [269]. The likelihood of a link between Aβ and NFT was reinforced by the demonstration of tau hyperphosphorylation nearby Aβ *in vitro* and *in vivo*. Abnormalities in Tau phosphorylation and cleavage can be induced by aggregated Aβ [270-272]. Neurofibrillary pathology and cortical neurodegeneration coexist in Tg models expressing both mutant human tau and APP as well as in Aβ injected tau mutant (P301L) animals [39,273,274]. However, NFT pathology is a major characteristic of neurodegenerative diseases that can take place in the absence of β-amyloidosis (e.g. frontal temporal dementia and parkinsonism-linked to chromosome 17, Pick’s disease, progressive supranuclear palsy, corticobasal degeneration and argyrophilic grain disease [275,276]. PS1 mutants presenting tau pathology of the FTD-type but no Aβ pathology might indicate that even so Aβ can trigger downstream phosphorylation of tau; tau can not initiate Aβ-deposition.

Ultimately, ADDLs caused a significant decrease in spine density observed without neuronal degeneration as represented in Fig. (**2**) [114,115,226]. Other research groups have reported similar observations. ADDLs can change synaptic connections by reducing the number of synaptic spines and altering both their morphology and motility [277]. Additionally, dendritic spine loss and transient spine elongation were surveyed in the presence of naturally secreted oligomers [244,278] and Aβ preparations containing oligomers [182,266].

In summary, the synaptic attack by ADDLs seems to offer a mechanistic link between various facets of early events in AD pathogenesis such as synapse loss [226], AD-type tau hyperphosphorylation [269], ROS formation [279], brain insulino-resistance [263] and selective neurodegeneration providing strong evidence for their involvement in memory impairment and ultimately dementia [114,115]. These findings suggested that ADDLs might act as gain-of-function ligands causing aberrations in signaling cascades related to LTP/LTD and memory but the cellular and molecular events underlying the toxic effects of Aβ oligomers is not entirely known. Only recently have the effects of Aβ oligomers on LTP and LTD been characterized, revealing the involvement of certain receptors and signaling pathways that will be discussed in the next section. 

The neurological properties of oligomers and their presence in AD brain coupled to the reversibility of memory loss in mouse models provide strong support for the hypothesis that AD is an oligomer-induced synaptic failure [19,101,103]. This hypothesis was significantly strengthened by the above demonstration that synapses are indeed the actual point of attack by oligomers where the oligomers produce their toxic effects by landing on particular post-synaptic receptors.

Synaptic binding of soluble Aβ could result from a “fatal attraction” of these species with metal ions (e.g. zinc and copper) that are highly concentrated at synapses triggering a toxic cascade [280,281]. Alternatively, it has also been proposed that globular and/or protofibrillar species could get inserted or bind to the lipid membrane bilayer instead of binding to membrane receptors. They would then create a pore that would confer an ion channel and induce downstream pathophysiological cellular responses resulting mainly from an interference with cellular ionic homeostasis [214,282,283]. 

Even though the soluble oligomeric species seem to be occupying a vanguard position, the relative impact of the plaque-deposited Aβ is still a focus of present research. We will conclude this section by saying that rather than asserting that only small soluble oligomers and not large insoluble deposits of Aβ represent the sole neurotoxic entity, a constant dynamic swap between the various forms should be pictured. In view of the severe abnormalities in synapses and neuronal processes associated to fibrillar deposits of Aβ, plaques and their surroundings might serve as reserve of soluble oligomeric Aβ that are not yet detectable at the neuropathological level [187,284,195]. It might then just be appropriate to attribute neuronal and synaptic dysfunctional activities to the soluble oligomeric species in the earliest stage of the AD process. 

## SYNAPTOTOXIC MECHANISMS TRIGGERED BY SOLUBLE Aβ OLIGOMERS OR ADDLs

As synaptic dysfunction appears to be one of the main pathophysiological theories of AD [19,257,285], it is important to find an etiological explanation for the alteration in synaptic signaling. In its initial appearance, the revised amyloid cascade claimed that synaptic dysfunction is one of the first presentations of AD pathology that is induced by Aβ toxicity but did not described the molecular pathway(s) by which Aβ attains its hypothetical synaptotoxic function [19]. Synaptic dysfunction in human and AD-Tg manifested themselves as deficits in neurotransmission and synaptic scaling [156,267], decreased synaptic density and deficits in synaptic plasticity [19,103,118,226]. Nevertheless, the described interference of ADDLs with synaptic transmission efficacy point to possible alterations in the intricate coordination of multiple specialized proteins involved either in synaptic vesicle trafficking or pre- and/or post-synaptic structure and plasticity [286]. Mechanisms underlying the effects of soluble Aβ on synapse dysfunction are not exactly known, although Aβ disturbs a plethora of cellular processes that ultimately could lead to decreased cell viability. It is likely that several degenerative pathways act simultaneously.

Comparison of gene expression in 3 different AD-Tg at 2 different ages reported a pretty good match between up- and down-regulated genes in these mice and what was previously reported in human AD brains [139,141]. The main category of genes that are commonly upregulated in these mice and AD, comprised genes involved in calcium and kinase signaling, growth factor and their receptor signaling, gene transcription, oxidative stress protection, lipid and cholesterol metabolism, cellular adhesion, lysosomal activation and inflammation. While, the key genes that are downregulated belong to the groups of genes involved in kinase and phosphatases signaling, trophic and growth factors, ion channels, protein metabolism, mitochondrial enzymes, transcription and translation and synaptic and vesicle transport. Down -regulation of particularly interesting genes, in view of the synaptic failure hypothesis, affected those encoding for the glutamate NMDA and AMPA receptors, potassium and calcium channels and αCamKII. Downregulation taking place later concerned genes of the Ras GTPase activation protein, the Ephrin receptors, GABA-A receptor, the synaptic vesicle transport proteins, the insulin-like growth factor binding protein and BDNF (for more details, see [141]). The changes in gene expression were exacerbated when large amounts of Aβ deposits were present but were already observable at times of soluble Aβ production. Similarly, five times more genes were modified in established AD compared to early AD [139]. These findings are compatible with some of the pathological processes previously reported including abnormal synaptic plasticity and structure.

Here we will focus on cellular processes that can be attributed to specific Aβ oligomeric forms and can be placed in the pathogenic cascade. Some mechanisms have recently emerged as depicted on Fig. (**3**). Further details can be found in the following reviews [156,235,287,288].

### Oxidative Stress

Oxidative stress is one of the strongest explanations for neuronal dysfunction related to alteration of neuronal signaling mechanisms and has been extensively described elsewhere [65,289,290]. Inhibition of LTP by soluble Aβ might result from the activation of stress kinases and oxidative stress mediators through NMDARs, mGluR5, TNFα death receptor or αv integrin [235,289]. Different Aβ oligomeric forms induced oxidative stress [279]. In addition, some synaptic protein genes present a highlighted susceptibility to oxidative stress damage and can be downregulated [291].

### Calcium Dyshomeostasis

Increase calcium influx is highly related to the presence of various forms of Aβ and has been extensively described (see reviews [292,293]). More precisely, Aβ peptides deposited in the dendritic arbor caused an increase in dendritic Ca^2+^ [294] and to local dendritic spines [278].

### Involvement of the Glutamate Receptors

The understanding of the functions exerted by the thousand proteins that constitute the synapse has just started with the definition of how some of them interact with each other in that unique neuronal compartment. Only a part of them have been well characterized making the comprehension of the exact molecular mechanism used by synaptically bound ADDLs even more difficult. One of the best functionally studied constituent of the synaptic machinery complex is the so called NMDA-R complex/MAGUK associated signaling complex (NRC/MASC) that contains ~185 proteins [295] with various possible functional interactions that are the cornerstone of synaptic plasticity. In such a refined system, it is comprehensible that any displacement or alteration (e.g. due to posttranslational changes or modified protein structure) of few of the constituents would have major detrimental effect to synaptic functions. 

Several studies have described altered glutamate receptors expression in the hippocampus of AD. Any alteration in expression and distribution would have severe consequences on synapse function and neuronal homeostasis because of their role in excitatory synaptic transmission. 

Expression of NMDAR subunits 1 and 2B, analyzed in the hippocampus throughout the progression of AD, was significantly decreased compared to control brains [296, 297]. In our hippocampal culture model, ADDLs triggered a decrease of both NR2B and NR1 at the plasma membrane level [226,259]. Soluble Aβ oligomer-induced LTP impairment necessitates the activation of group I metabotropic glutamate receptor activation [234].

Even so we found association between ADDLs and NMDAR [226], we do not know if it is through a direct interaction. However prevention of ADDL-triggered dendritic spine loss by memantine, an uncompetitive NMDAR open-channel antagonist [226] and interference with the NMDAR signaling pathway in the presence of the naturally secreted oligomers [278] strongly suggests the contribution of NMDAR in soluble Aβ synaptotoxicity. Memantine is used to treat the symptoms of AD and works by decreasing Aβ “hyperactivation” of NMDAR [298] therefore providing memory benefits possibly by reducing ADDL-induced calcium influx and related downstream signaling cascade as well as Arc overexpression. Excessive activation of NMDAR was reported to produce spine loss [299] which would agree with the idea that soluble Aβ by “overexciting” the NMDAR set off spine loss described above.

It is thought that the release and uptake of the neurotransmitter glutamate is dysfunctional in AD, leading to an elevated neuronal influx of calcium triggering downstream neurotoxic pathways, a neurodegenerative cascade that can partially be alleviated by memantine. 

In an elegant study, it was proposed that activation by Aβ of the α7nAchR and downstream tyrosine phosphatase STEP is required to elicit the internalization of NMDAR in cultured hippocampal neurons [265]. The activation of STEP is responsible for the dephosphorylation of NR2B and the inhibition of Fyn, another tyrosine kinase which can phosphorylate NR2B. These events drive the decrease of NMDAR surface receptors both by interfering with endocytic pathway and blockage of the exocytosis of new NMDAR at the surface [300]. 

Aβ species can selectively bind with picomolar affinity to α7 nAChR [301], directly modulate nAChRs [302], block the α7 nAChR mediated responses [303] and activate mitogen-activated protein kinase (MAPK) cascade *via *α7 nAChRs [304]. In addition, nicotine and nicotinic agonists have effects against Aβ toxicity [305]. However, blockage of a7nAchR by α-bungarotoxin was not complete suggesting the possible involvement of additional receptor mechanisms in Aβ synaptotoxicity. 

#### AMPARs

Aβ42 attenuates AMPAR function [306], reduces litude and frequency of AMPAR mediated excitatory postsynaptic currents in hippocampal CA1 neurons and reduces the opening probability and open time of the channel [266,267]. Both AMPAR and NMDAR currents were downregulated in hippocampal slices incubated with a virus containing hAPP [59]. AMPAR subunit GluR2 is significantly decreased in AD prior to NFT formation [307]. GluR2 is critical for keeping AMPAR impermeable to calcium ions; therefore its decrease considerably affects synaptic function and neuronal viability. Data on GluR1 levels in AD brains did not show correlation with disease severity [308].

Furthermore, AMPAR internalization occurs downstream of group 1 mGluR activation [309], and could potentially explain the complete decay of short-term potentiated responses observed after Aβ-oligomer exposure in LTP experiments [116,118]. The endocytosis of AMPAR under Aβ exposure uses signaling pathways of LTP such as p38MAPK and calcineurin/PP2B [266]. Activity of PP2B seems also required for NMDAR withdrawal [265]. 

#### mGluRs 

Elongation of spines is typically associated with a moderate increase in intracellular calcium levels resulting from intracellular store release triggered by activation of metabotropic receptors such as group 1 mGluRs [310]. Activation of group 1 mGluRs is critical for soluble Aβ induced LTP impairment, and provides a reasonable link between the observed spine elongation and ADDL exposure.

Activation of mGluR have been shown to rapidly increase the aggregation of polyribosomes and to increase spine size [310]. Involvement of the mGluRs in the Aβ-mediated reduction of glutamatergic transmission has been postulated, while activation of this synaptic transmission type is increased through activation of presynaptic α4β2 nAchRs [311]. 

### NMDAR-Associated EphB Receptor

EphB receptor tyrosine kinases represent another synaptic receptor essential for hippocampal synaptic plasticity and proper postsynaptic specialization [312]. Its activation induces an extracelluar interaction with NMDAR1 [313] promoting enhanced NMDAR Ca2+ influx and clustering of NMDAR with αCaMKII recruited to the PSD for proper synaptic transmission [313]. Interestingly, mice expressing a truncated form of EphB2 without the tyrosine kinase site presented lower spine density and longer spine length [314].

EphB2 activation promotes the synaptic recruitment of the Rho-GEF kalirin and the activation of Rac1 and its effector PAK, a downstream effector of the Rho/Rac family of small GTPases [315]. The activation of PAK regulates signaling cascades that cause actin rearrangements and spine morphogenesis [316] and its inhibition has been associated with severe mental retardation and memory deficits [317]. Neuronal exposure to Aβ oligomers causes a direct loss in neuronal PAK signaling suggesting that the loss of surface NR1 by short-term ADDL treatment may then instigate the reduction of EphB2 surface density. Loss of these receptors at the synapse is believed to induce defects in the PAK signaling pathway, presumably through changes in synaptic recruitment of kalirin and Rac1. Changes in this pathway have been proposed to be responsible for the drebrin loss and aberrant spine structure observed with ADDL treatment [317]. 

One might speculate that this series of events is responsible for the loss of connectivity responsible for memory deficits in the AD brain, as NMDAR, AMPAR and EphB receptors are important for plasticity and spine structure. 

### Disorganization of the PSDs 

Concomitantly to the reported changes in memory-essential receptors (see above), Aβ has been shown to instigate the degradation of PSD95, a “multi-partners” molecule binding scaffolding and cytoskeletal proteins as well as adhesion and signaling molecules and glutamate receptors [248]. The PSD95 complex represents a network of molecular components providing compartmentalization and specificity for both temporal and spatial organization in signaling pathways [318]. PSD95 regulates AMPAR and NMDAR receptor trafficking [319,320] which would explain why its soluble Aβ-induced degradation is concomitant to lower surface expression of these glutamate receptors [264].

### Corruption of the F-Actin Cytoskeleton Complex (Arc, Drebrin and Cofilin) and Involvement of RhoGTPases Signaling Cascade (Rac/Rho/cdc42, PAK, LIMK)

**Fig. (3):**
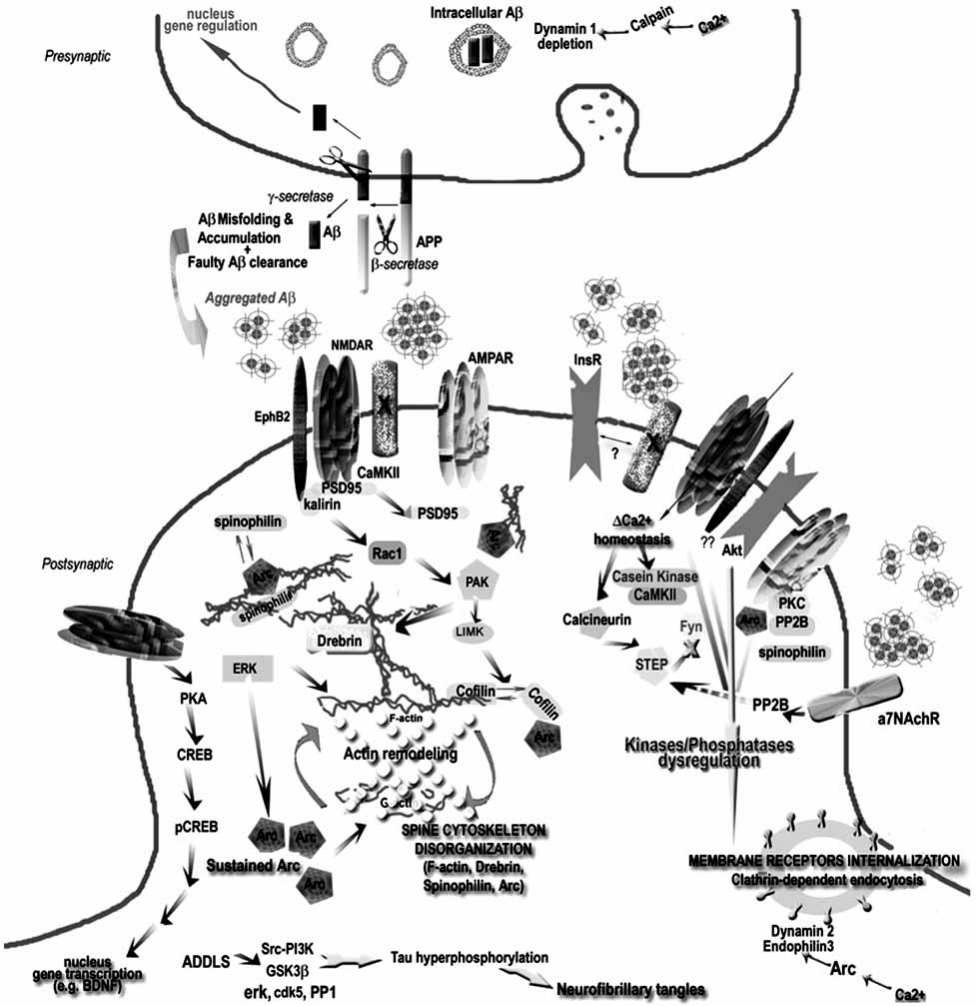
Summarized neurotoxic mechanisms of synaptically bound ADDLs. Proposed mechanistic pathway for synaptically-bound ADDLs implicated in the induction of aberrations in dendritic spine composition and morphology and how this mechanism is related to synaptic dysfunction and connectivity loss in AD. Aβ generated by cleavage of APP can accumulate and adopt oligomeric conformations of the ADDL-type that will bind to a postsynaptic membrane receptor (protein X) not yet identified but putatively located in the NMDA-R complex triggering Ca2+ influx followed by various changes in kinases/phosphatases activity that lead to: 1) reorganization of memory-essential receptors (NMDA-R, AMRA-R, EphB and InsR) from the cell surface most probably due to faulty receptor endocytosis. Various mechanisms have been proposed: one of them implicated Arc overexpression in the withdrawal of AMRA-R, while another suggested that NMDA-R endocytosis results from ADDL-induced activation of the a7nAchR and participation of signaling molecules such as STEP and Fyn. Alterations in dynamin 2 and endophilin, an enzyme involved in endocytic machinery controlling receptor turnover, has also been proposed. 2) deregulation of actin cytoskeletal dynamics which might result from the activation of Arc. At this level, rearrangement inside the actin-binding protein network, which is partially composed of Arc, Drebrin, Cofilin and Spinophilin, takes place disrupting the “spine morphology motor” and therefore proper synaptic plasticity. PAK and RhoGTPases activities are believed to play a major role in the actin cytoskeleton dynamics, its reduced activity leading to loss of drebrin and activation of cofilin, an actin-depolymerizing molecule. 3) Modifications of the microtubule network examplified by the hyperphosphorylation of tau and the alterations in tubulin and MAP2 (not represented here). 4) Possible interference with expression of survival and “killer” genes through the CREB pathway. For clarity purpose, other possible factors (e.g. oxidative stress, mitochondrial dysfunction, presynaptic neurotransmitter release) that might be implicated in synaptic receptor expression and spine morphology have been omitted.

Morphological plasticity of spines depends on an intact spine cytoskeleton that is characterized by a dense meshwork of actin-containing microfilaments (review in [321] and reference therein) and many cytoskeletal associated proteins that cross-link actin filaments to the plasma membrane and to the PSD itself [322]. The level of F-actin in the spine is determined by an equilibrium between polymerized and depolymerized actin (Fig. **3**), a phenomenon highly controlled by actin regulatory and binding proteins (e.g. ADF/cofilin, profilin) that are synchronized by the PSD-network [323]. Dynamic changes in F-actin are linked to postsynaptic NMDAR [324] and determine the capacity of the spines to go through morphological changes [325]. Key pathways pairing actin remodeling and NMDAR have been recently reviewed [312]. As mentioned above, Aβ-induced changes in NMDAR and EphB2 receptors expression at the synaptic surface, without changing their total amount, may cause downstream signaling defects in the PAK pathway. Some of these signaling modifications may elicit actin rearrangements, therefore antagonizing spine motility and impairing synaptic plasticity leading to the unconventional presentation of dendritic spine structure and density observed with prolonged ADDL treatment.

A main pathway undergoes regulation of RhoA and Rac1 (small RhoGTPases, [327]), key mediators of synaptic plasticity [327]. Rac1 plays a main role in the regulation of F-actin polymerization, implicating it in the maintenance and reorganization of dendritic structures, both at the level of their stability and motility [326,328]. Rac1 activation promotes spine formation filopodia-like (elongated spines without growth of spine heads) to the detriment of normal spines while its inhibition is linked to decreased spine density. To the contrary, RhoA activation leads to a reduction of spine length and density while its inhibition results in motility of new spines [326,329,330]. Considering the spine morphology and density changes observed in soluble Aβ oligomer-treated neurons, it seems that both paths could occur simultaneously throughout the dendrites and maybe differentially among dendritic spines. This would, at least for a determined time, lead to the mixture of spine withdrawal and spine extension we have observed after ADDL incubation of hippocampal neurons [226]. Activation of Rac1 has been shown to occur after exogenous application of Aβ [104,331] and is observed in AD and AD-Tg brains [332]. PAK, a Rac1 effector, and ADF/cofilin, a downstream actin depolymerizing protein were independently thought to be implicated in AD [317]. Downregulation of cofilin occurs after its phosphorylation by PAK/LIMK pathway [333,334], therefore reduction of PAK and LIMK like what is observed in AD and AD-Tg would upregulate cofilin and disorganize actin complex. In fact, PAK seems to play an essential role in the synaptotoxic cascade of soluble Aβ oligomers because its overexpression prevents Aβ oligomer-induced drebrin loss while its inhibition causes drebrin loss and definite memory deficits [317]. Rac1 was also reported to be essential in the Aβ-induced signaling cascade that leads to the generation of ROS in astroglioma cells [335]. Rho is in charge of the regulation of clustering between F-actin and actin-binding proteins such as spinophilin and neurabin. It is therefore expected in the case of Aβ synaptotoxcity that alterations in synaptic protein stoechiometry between F-actin, drebrin, Arc, spinophilin and cofilin would occur. 

Interestingly, we have reported that soluble Aβ oligomers targeted synapses in cultured rat hippocampal neurons where they triggered sustained overexpression of the immediate-early gene Arc [114,115,122]. Arc protein has an essential role in long term memory formation and synaptic plasticity [223,336] and is known to regulate both the state of F-actin and microtubule network by interacting with MAP2. We have predicted that ADDLs by increasing Arc expression would interfere with glutamate receptor expression at the surface and cause aberrations in spine shape [122]. This prediction has largely been confirmed [226,259,265,266]. Moreover, Arc has been granted a function in AMPAR trafficking by promoting receptor endocytosis after interaction with two proteins of the endocytic vesicle membrane (dynamin and endophilin) [337]. Compatible with the deleterious effect of Aβ at synapses, Arc overexpression downregulated AMPAR surface expression [338], induced a reduction of AMPAR-mediated synaptic transmission [339] and blocked the homeostatic increase in AMPAR function [340]. Even so NMDAR downregulation from the cell surface predicts a decrease in Arc mRNA translation at the synapse, Arc expression might be deregulated and elevated when AMPAR are withdrawn from the membrane because they normally serve as a negative regulator of Arc transcription [341]. We do not know yet if Arc could disrupt cycling of other synaptic receptors required for synaptic plasticity.

Spinophilin bundles actin, but also facilitates synaptic transmission by recruiting protein phosphatase to the synapse to modulate glutamate receptor activity [342]. The impact of the unbalanced regulation inside the postsynaptic actin-regulatory machinery (loss of drebrin associated to increase dephosphorylated cofilin) and its role in synaptic dysfunction characteristics of AD and DS was nicely reviewed elsewhere [184]. This drebrin/actin-regulatory proteins/F-actin complex is highly regulated by NMDAR activity [325]. 

### Tyrosine Kinases and MAP Kinases

Evidence also suggested that Aβ oligomers bind to neuronal membrane proteins [119,121] resulting in activation of kinase signal transduction pathways [101,343,234]. Aβ was shown to regulate the function of PKA, calcineurin and CREB [344,345]. 

Aβ1-42, under fibrillar or soluble conformation, can induce a sustained activation of ERK1/2 signaling pathway which can then lead to hyperphosphorylation of Tau [271,346,347]. In the case of soluble Aβ oligomers (AβOs), caspase-3 activation following the stimulation of the ERK1/2 signaling pathway leads to proteolytic cleavage of tau [272] without synapse degeneration. This suggests that oligomers can induce microtubule damage.

Other pathways involving PKC [348], JNK, cdk5 and p38 MAPK [234] have been proposed for Aβ-mediated toxicity implicating possibly different signaling pathway activation that might very well be due to the use of different conformational Aβ species or particularities in the experimental protocols used.

Other studies implicated Fyn, a src-family tyrosine kinase, in the toxicity of soluble Aβ oligomers. Initial demonstrations elegantly pointed out to Fyn as an essential element in synaptotoxicity as knock-out of Fyn in cells and hAPP-Tg mice protects against Aβ toxicity [101,343] while Fyn overexpression exacerbated the loss of synaptic markers and subsequent memory dysfunction [343,349]. 

### Insulin Pathway

Soluble Aβ oligomers have very recently been shown to interfere with insulin receptors present in the brain providing a basis for CNS insulin resistance in AD [115,263]. Mechanisms of ADDL-induced InsR withdrawal from the cell surface have not been described yet but it is becoming clear that soluble Aβ greatly reduces the responsiveness of the neurons to insulin. Concomitantly, a report showed that naturally secreted oligomers interfere with ERK/MAPK, CaMKII and PI3K/Akt, effects that are supposedly resulting from the binding of Aβ oligomers to the InsR [350], if so it is speculated that insulin would provide protection against some Aβ-induced synaptotoxic effects. Competition between Aβ and insulin for InsR binding site results in a decrease in InsR-mediated signaling [263,350-352]. Indeed, insulin can protect against Aβ cytotoxicity in hippocampal cell cultures [353] and can regulate the endocytosis of AMPAR [354].

### Other Events Relevant to Synaptotoxicity 

Alterations in the synaptic vesicle trafficking were reported in neurons exposed to Aβ species [355]. Briefly, Aβ triggers an increase in intracellular calcium most probably through the activation of voltage-gated calcium channels. This activates subsequently the calpain enzyme which has dynamin-1 for substrate. Dynamin-1 is a GTPase protein which participates in the exo/endocytosis mechanisms by helping the recycling of synaptic vesicles from the plasma membrane after neurotransmitter release [356]. Aβ hence accentuates neurotransmitter release.

At the same time, Aβ can also induce proteasome dysfunction by inhibiting the ubiquitin C-terminal hydrolase [357] which could in turn lead for example to accumulation of tau [358] and improper turnover of PSD protein [264] thus it is legitimate to hypothesized that the various events described above will ultimately lead to abnormal PSD composition. 

## PERSPECTIVES – CONCLUSIONS

While the cellular and molecular events underlying the toxic effects of Aβ oligomers are not entirely known, it is evident that soluble Aβ oligomers have synaptotoxic effects and contribute to the increasing body of knowledge regarding the molecular basis for memory loss in AD. We proposed that the selective targeting of ADDLs to the post-synaptic sites creates an inappropriate organization of the cytoskeletal and scaffolding PSD network. Association and activation of signaling molecules at the PSD are disrupted which in turn alter the modulation of actin-based spine morphology and receptor distribution contributing to early cognitive deficits of AD. Synaptic disruption by ADDLs might even derive from modulations of multiple signaling pathways and cytoskeletal reorganization. Gradually more understanding of the molecular biology of early AD will provide new therapeutic targets.

## Figures and Tables

**Fig. (1):**
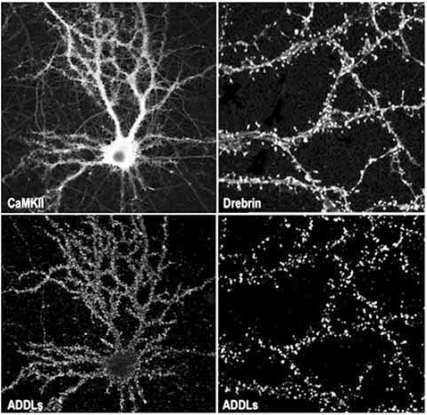
Upper: Neurons and more precisely dendritic spines were visualized on a cultured hippocampal neuron by labeling with anti-CaMKII and anti-drebrin antibodies. Lower: immunofluorescence for the ADDL bound onto the hippocampal cells was revealed using an anti-oligomer specific antibody. Labeling reveals that ADDL distributed along dendritic arbors particularly attack dendritic spines (as demonstrated by high degree of colocalization between drebrin and ADDLs).

**Fig. (2):**
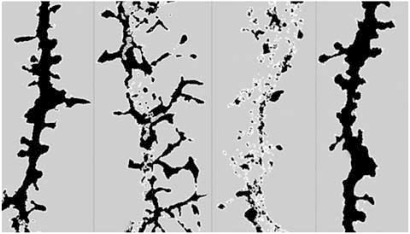
ADDL binding to dendritic spines causes time-dependent changes in dendritic spine morphology and density as illustrated here by drebrin immunoreactivity, a dendritic spine marker. Computer-derived profile outlines were generated from a z-stack reconstruction of single dendritic branche imaged from a confocal scanning of drebrin-immunolabeled neurons. The treatment conditions represented here were in the following order ADDL 500nM for 5min, ADDL 500nM for 6hrs, ADDL 500nM for 24hrs and Vehicle for 24hrs. The same threshold setting was applied under the treatment conditions and show that both the dendritic spine density was decreased after ADDL treatment and that some spines are abnormally long after a prolonged ADDL treatment.
